# Supplementary material for: Optimized phenotype definitions boost GWAS power
Source: PLoS Comput Biol. 2026 Jul 1;22(7):e1014431. doi: 10.1371/journal.pcbi.1014431 (PMC13340767; doi:10.1371/journal.pcbi.1014431)
Supplement: S1 Text — Details of UK Biobank data processing and simulation parameters. (DOCX) [file pcbi.1014431.s001.docx]

Supplementary Materials

Processing of UK Biobank data

To begin, we selected a cohort using only White British individuals to reduce the effects of population structure on our analysis. Next, we removed individuals whose data were flagged for various reasons as being potentially flawed or erroneous. Specifically, we removed individuals whose genetic sex was mismatched with their self-reported sex, individuals who were outliers for heterozygosity or missingness (defined by the UK Biobank using the outlier detection algorithm, *abberant* ), individuals with ten or more third-degree relatives in the UK Biobank, individuals with sex chromosome aneuploidy, and we restricted to individuals used in the computation of genetic principal components by the UK Biobank. Finally, we restricted to individuals with diagnosis data available, as described below.

We gathered phenotypic data for this cohort using the International Statistical Classification of Diseases and Related Health Problems, 10th revision (ICD-10) codes. In the UK Biobank, these can be obtained from six different data fields, and we included them all. These fields are hospital inpatient ICD-9 codes, hospital inpatient ICD-10 codes, self-reported non-cancer illness codes, primary cause of death, secondary cause of death, and general practitioner outpatient diagnoses. We used mappings provided by the UK Biobank to convert each coding to ICD-10. Only codes with at least 100 observations were retained. Applying the above QC and filtering procedure resulted in 1238 binary phenotypes and 342,350 samples. To save computation time, we restricted our analysis to HapMap3 SNPs, resulting in 1,166,145 SNPs in our final dataset.

All analyses of real data used Plink 2 for GWAS, with the top 10 genetic principal components, age, and sex used as covariates. We used SumHer to estimate genetic covariances between feature and target phenotypes. Following the advice of Speed and Balding , we estimated genetic correlation with the LDAK-Thin model and heritability using the BLD-LDAK model, using tagging files provided at <https://dougspeed.com/pre-computed-tagging-files>. As a final QC step, we removed all estimates that were outside the range of possible true values (i.e. heritability outside [0, 1], genetic correlation outside [-1, 1], or standard error greater than two). This meant that each MaxGCP phenotype had a slightly different subset of the feature traits from which to select.

Details of the simulation

Realistic effect sizes and correlations among phenotypes are essential for evaluating MaxGCP. The PhenotypeSimulator R package is designed for this purpose, and it can simulate phenotypes using real genetic data. We used this package to generate phenotypes with known effect sizes and correlations. To construct the cohort for this simulation, we randomly sampled 10,000 (White British, QC-passing) individuals and 100,000 (imputed, HapMap3, QC-passing) variants from the UK Biobank.

PhenotypeSimulator has many tunable parameters that control the genetic and phenotypic properties of the simulated phenotypes. Heritability, genetic correlation, and phenotypic correlation were the only relevant parameters for this study. We took a two-step approach to setting these parameters. First, we picked two sets of values that we believed to be realistic. Second, to evaluate the sensitivity of our results to these choices, we individually varied each parameter while keeping the others constant at realistic values.

To determine realistic settings for these parameters, we estimated the heritability, genetic correlation, and phenotypic correlation of real diseases in the UK Biobank. For convenience, we used only the 20 most common ICD-10 codes in our QC-passing White British cohort. After estimating these values in real data and performing QC on the results, we found that the median heritability was 0.05, the median genetic correlation was 0.49, and the median phenotypic correlation was 0.10. We sought to simulate phenotypes that approximately matched these values. PhenotypeSimulator input parameters define distributions, not the actual values of the resulting phenotypes. Accordingly, we adjusted the PhenotypeSimulator parameters so that the correlations match (S1 Fig). We set heritability to 0.01, genetic correlation to 0.5, and phenotypic correlation to 0.0.

This simulation resulted in individual-level genotypes, phenotypes, and phenotypic genetic components. The format and structure of these data are very similar to real data, with a few notable exceptions. First, the simulated variant effect sizes are independent of one another, and there is no relationship between variant effect size and LD. This means that LDSC-like methods for heritability estimation are inapplicable. Second, we did not generate any covariates or confounders, as they are not relevant to the current analysis. Our simulation is intentionally much smaller than the UK Biobank dataset, with fewer variants, samples, and phenotypes. Our goal was not to recreate the UK Biobank in simulation, but to evaluate the feasibility of MaxGCP, the correctness of our definitions, derivation, and implementation, and whether MaxGCP achieves its aims.

We simulated phenotypes with these settings and computed the actual heritability, genetic correlation, and phenotypic correlations. The distributions of these values showed good correspondence to their distributions in real data (S1 Fig). MaxGCP depends on correlations and genetic signals to function, so it is expected to perform better with larger values of these parameters. We set the values of the simulation parameters conservatively so that the resulting distributions have similar or smaller means than we observe in real data. Overall, these results suggest that the simulation is reasonably similar to real data, albeit with known genetic effects.
