# Supplementary figures and images for: Optimized phenotype definitions boost GWAS power
Source: PLoS Comput Biol. 2026 Jul 1;22(7):e1014431. doi: 10.1371/journal.pcbi.1014431 (PMC13340767; doi:10.1371/journal.pcbi.1014431)

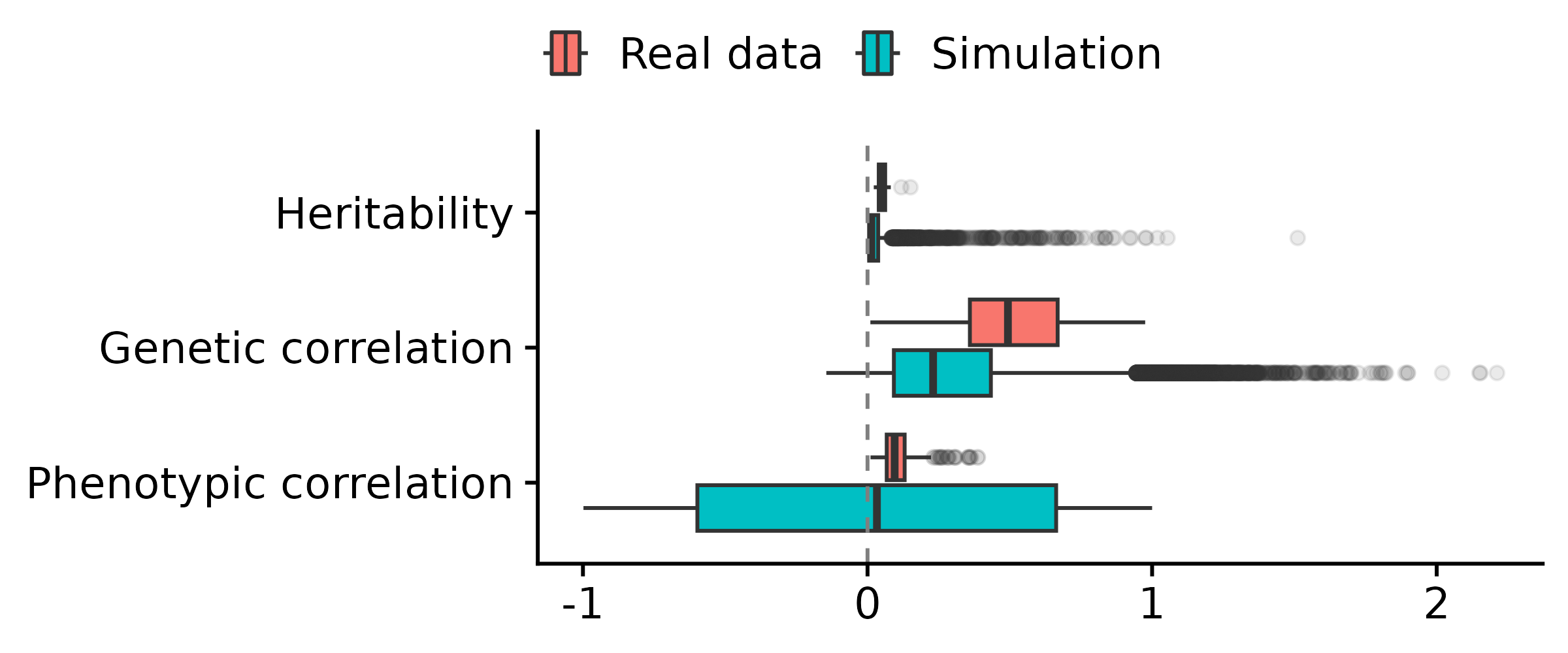

Supplement: S1 Fig — Shown is a comparison between simulation and the 20 most common ICD-10 codes in the UK Biobank, in terms of heritability, genetic correlation, and phenotypic correlation. The simulated phenotypes were generated using PhenotypeSimulator with expected heritability 0.01, genetic correlation 0.5, and phenotypic correlation 0. These simulation parameters were chosen so that the resulting simulation is similar or more conservative than the real data shown. (TIF) [file pcbi.1014431.s002.tif]

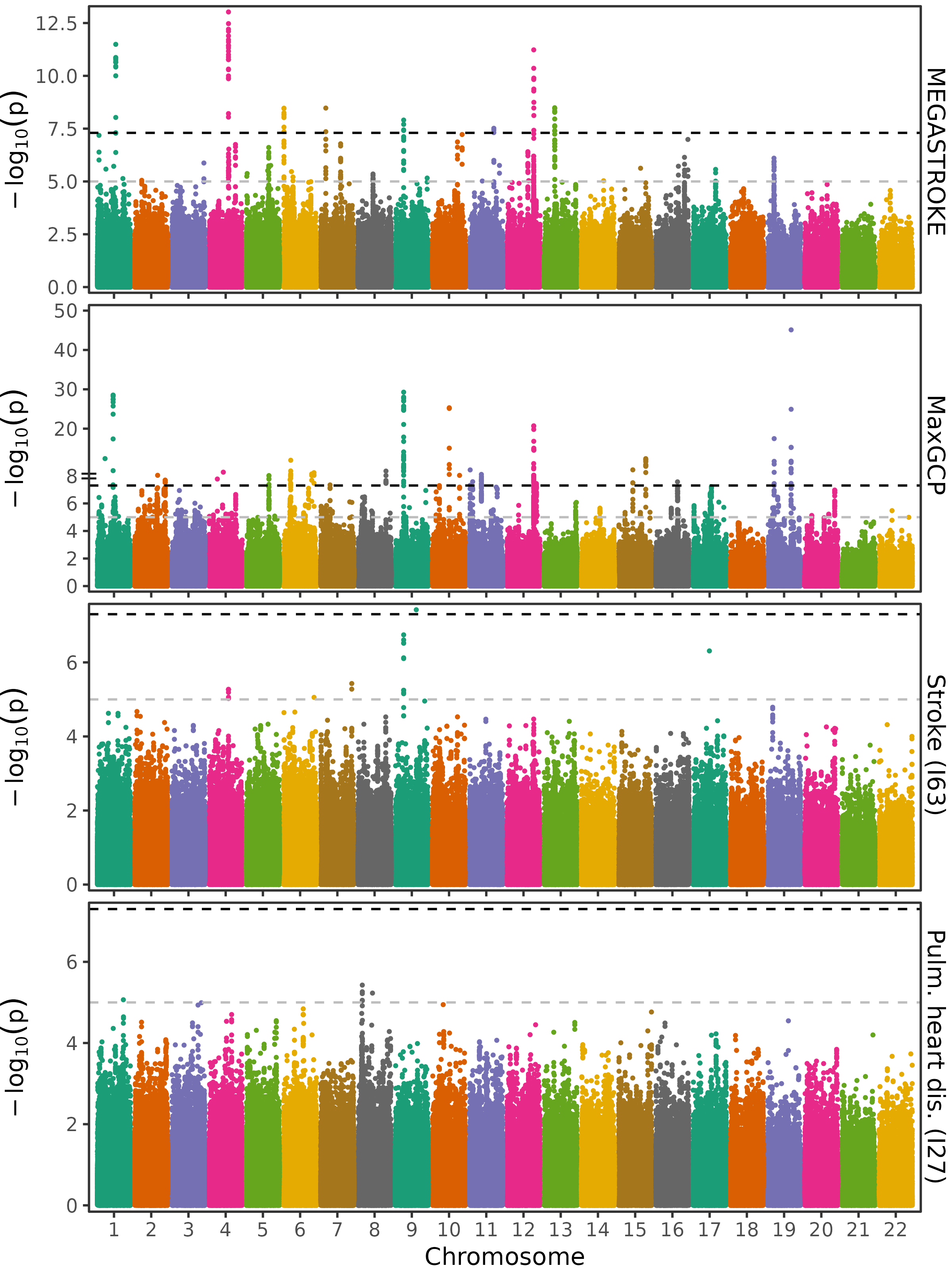

Supplement: S2 Fig — Shown are GWAS summary statistics for the target, MaxGCP phenotype, naive phenotype, and top feature for the MEGASTROKE “Any stroke” phenotype. MEGASTROKE shows the MEGASTROKE “Any stroke” GWAS summary statistics. MaxGCP shows the corresponding MaxGCP phenotype. Stroke shows the naive equivalent phenotype, defined as a single occurrence of any ICD-10 code starting with I63. The final facet shows GWAS summary statistics for pulmonary heart disease, the top feature in MaxGCP besides stroke itself. (TIF) [file pcbi.1014431.s003.tif]

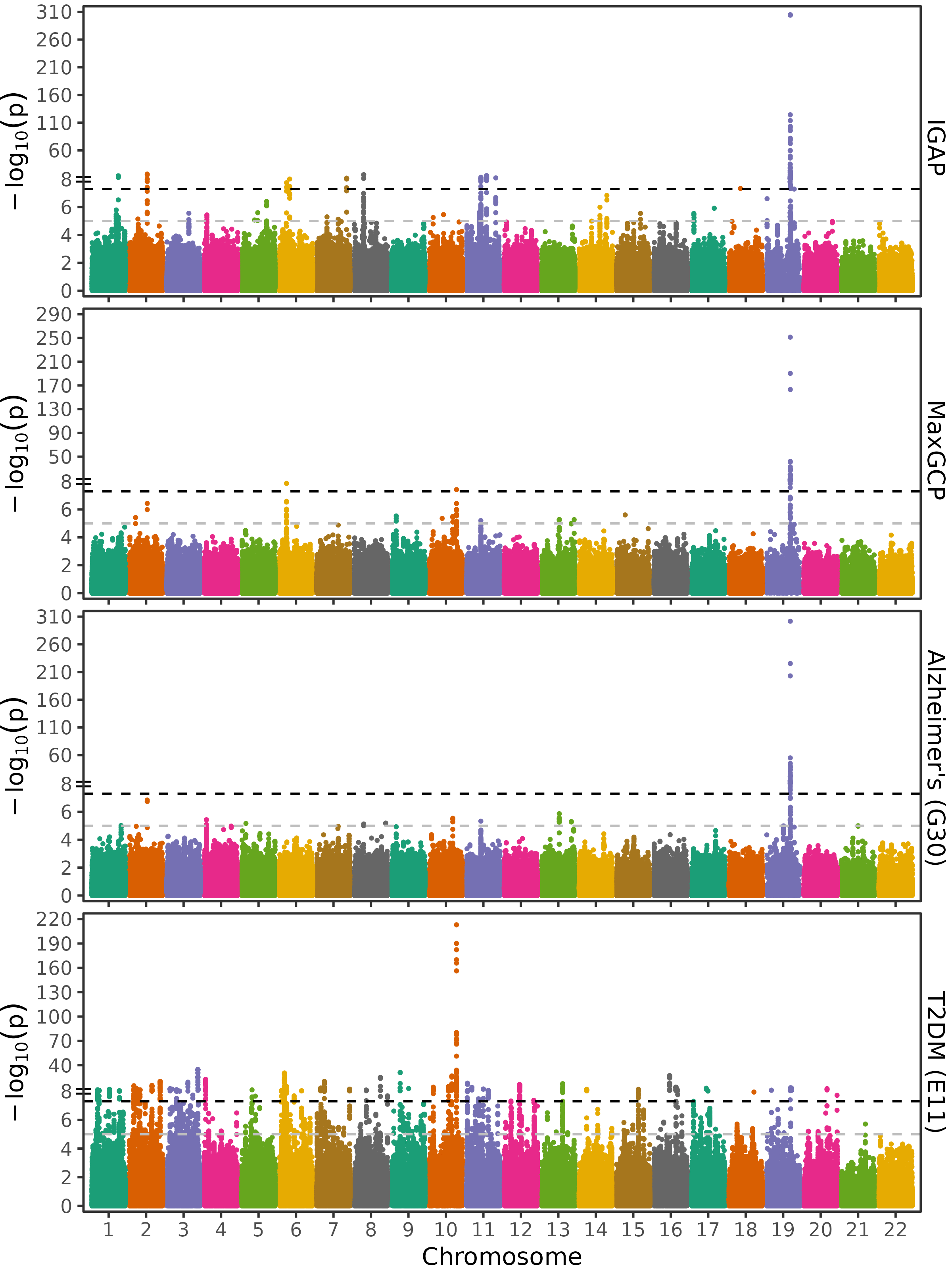

Supplement: S3 Fig — Shown are GWAS summary statistics for the target, MaxGCP phenotype, naive phenotype, and top feature for the IGAP Alzheimer’s disease phenotype. IGAP shows the IGAP GWAS summary statistics directly. MaxGCP shows the corresponding MaxGCP phenotype. Alzheimer’s shows the naive equivalent phenotype, defined as a single occurrence of any ICD-10 code starting with G30. The final facet shows GWAS summary statistics for type 2 diabetes mellitus, the top feature in MaxGCP besides Alzheimer’s disease itself (ICD-10 code G30). (TIF) [file pcbi.1014431.s004.tif]
