# Supplementary material for: Optimized phenotype definitions boost GWAS power
Source: PLoS Comput Biol. 2026 Jul 1;22(7):e1014431. doi: 10.1371/journal.pcbi.1014431 (PMC13340767; doi:10.1371/journal.pcbi.1014431)
Supplement: S1 Table — Each reported metric is the median of per-phenotype values. A p-value cutoff of 0.05 was used to declare variants as predicted positives or negatives, and associations were compared to those correctly identified by the genetic component GWAS, making it perform perfectly by construction. Methods marked by an asterisk (*) are unobservable in real data. (DOCX) [file pcbi.1014431.s005.docx]

**S1 Table. GWAS performance of each phenotype definition in simulation.** Each reported metric is the median of per-phenotype values. A p-value cutoff of 0.05 was used to declare variants as predicted positives or negatives, and associations were compared to those correctly identified by the genetic component GWAS, making it perform perfectly by construction. Methods marked by an asterisk (*) are unobservable in real data.

| **Method** | **Sensitivity** | **Specificity** | **Precision** | **F1** | **AUROC** |
| --- | --- | --- | --- | --- | --- |
| Genetic component* | 1.00 | 1.00 | 1.00 | 1.00 | 1.00 |
| Optimal linear* | 0.72 | 0.96 | 0.92 | 0.81 | 0.93 |
| MaxGCP (optimal) | 0.69 | 0.96 | 0.92 | 0.79 | 0.91 |
| MaxGCP (noisy) | 0.47 | 0.94 | 0.84 | 0.60 | 0.82 |
| Naive | 0.28 | 0.96 | 0.80 | 0.41 | 0.72 |
| Binary* | 0.37 | 0.96 | 0.85 | 0.52 | 0.79 |
| Liability* | 0.55 | 0.96 | 0.91 | 0.68 | 0.89 |
