# Supplementary material for: Optimized phenotype definitions boost GWAS power
Source: PLoS Comput Biol. 2026 Jul 1;22(7):e1014431. doi: 10.1371/journal.pcbi.1014431 (PMC13340767; doi:10.1371/journal.pcbi.1014431)
Supplement: S2 Table — Shown are selected features for each target phenotype. Each MaxGCP phenotype had a slightly different set of input features due to the QC process, in which noisy genetic covariance estimates were removed. Genetic correlations and standard errors were estimated using SumHer and the LDAK-Thin model. (DOCX) [file pcbi.1014431.s006.docx]

**S2 Table. MaxGCP coefficients for each target phenotype.** Shown are selected features for each target phenotype. Each MaxGCP phenotype had a slightly different set of input features due to the QC process, in which noisy genetic covariance estimates were removed. Genetic correlations and standard errors were estimated using SumHer and the LDAK-Thin model.

| **Target** | **Feature** | **MaxGCP** | $\boldsymbol{r}_{\boldsymbol{G}}$ | **SE** |
| --- | --- | --- | --- | --- |
| Any | Cerebral infarction | 2.467 | 0.847 | 0.160 |
|  | Other cerebrovascular | 1.665 | 0.745 | 0.146 |
|  | Acute ischemic heart disease | 3.589 | 0.679 | 0.147 |
|  | Other heart disease | 0.503 | 0.635 | 0.085 |
|  | Ischemic heart disease | 0.311 | 0.607 | 0.045 |
|  | Essential hypertension | 0.488 | 0.584 | 0.043 |
|  | Pulmonary heart diseases | 4.865 | 0.538 | 0.293 |
|  | Acute myocardial infarction | 0.898 | 0.511 | 0.053 |
|  | Type 2 diabetes mellitus | 0.901 | 0.389 | 0.041 |
|  | Obesity | 0.329 | 0.310 | 0.041 |
|  | Triglycerides | -0.003 | 0.193 | 0.035 |
|  | Type 1 diabetes mellitus | 1.988 | 0.189 | 0.142 |
|  | HbA1c | -0.159 | 0.179 | 0.037 |
|  | Glucose | -0.044 | 0.179 | 0.047 |
|  | Creatinine | 0.010 | 0.066 | 0.032 |
|  | Apolipoprotein B | 0.024 | -0.019 | 0.044 |
|  | Lipoprotein A | -0.031 | -0.045 | 0.081 |
|  | Cholesterol | 0.109 | -0.143 | 0.046 |
|  | HDL cholesterol | 0.003 | -0.199 | 0.035 |
| Cardioembolic | Pulmonary heart diseases | 9.772 | 0.715 | 0.725 |
|  | Cerebral infarction | 3.281 | 0.682 | 0.204 |
|  | Other heart disease | 0.248 | 0.397 | 0.145 |
|  | Ischemic heart disease | 0.467 | 0.349 | 0.080 |
|  | Acute ischemic heart disease | 2.124 | 0.318 | 0.171 |
|  | Essential hypertension | 0.275 | 0.286 | 0.075 |
|  | Other cerebrovascular | 0.025 | 0.249 | 0.179 |
|  | Acute myocardial infarction | 0.237 | 0.230 | 0.081 |
|  | Type 2 diabetes mellitus | 0.527 | 0.176 | 0.061 |
|  | Glucose | 0.009 | 0.142 | 0.060 |
|  | Obesity | 0.039 | 0.112 | 0.071 |
|  | HbA1c | -0.088 | 0.083 | 0.053 |
|  | Creatinine | 0.032 | 0.062 | 0.054 |
|  | Type 1 diabetes mellitus | 0.221 | 0.047 | 0.210 |
|  | Triglycerides | -0.058 | 0.017 | 0.054 |
|  | Apolipoprotein B | -0.013 | -0.057 | 0.065 |
|  | HDL cholesterol | -0.013 | -0.064 | 0.051 |
|  | Lipoprotein A | -0.042 | -0.097 | 0.148 |
|  | Cholesterol | 0.107 | -0.100 | 0.066 |
| Ischemic | Cerebral infarction | 2.770 | 0.844 | 0.148 |
|  | Other cerebrovascular | 1.528 | 0.675 | 0.135 |
|  | Acute ischemic heart disease | 3.737 | 0.648 | 0.143 |
|  | Ischemic heart disease | 0.324 | 0.567 | 0.045 |
|  | Other heart disease | 0.400 | 0.552 | 0.078 |
|  | Essential hypertension | 0.471 | 0.533 | 0.040 |
|  | Acute myocardial infarction | 0.946 | 0.485 | 0.052 |
|  | Pulmonary heart diseases | 4.036 | 0.430 | 0.254 |
|  | Type 2 diabetes mellitus | 0.896 | 0.365 | 0.040 |
|  | Obesity | 0.267 | 0.266 | 0.040 |
|  | Type 1 diabetes mellitus | 2.409 | 0.199 | 0.162 |
|  | Triglycerides | 0.003 | 0.183 | 0.032 |
|  | HbA1c | -0.157 | 0.181 | 0.036 |
|  | Glucose | -0.043 | 0.180 | 0.044 |
|  | Creatinine | 0.015 | 0.068 | 0.031 |
|  | Apolipoprotein B | 0.038 | -0.012 | 0.043 |
|  | Lipoprotein A | -0.027 | -0.014 | 0.053 |
|  | Cholesterol | 0.095 | -0.132 | 0.044 |
|  | HDL cholesterol | 0.010 | -0.185 | 0.033 |
| Small vessel | Obesity | 1.435 | 0.919 | 1.390 |
|  | Type 1 diabetes mellitus | 7.876 | 0.767 | 1.197 |
|  | Pulmonary heart diseases | 4.953 | 0.733 | 1.290 |
|  | HbA1c | -0.052 | 0.565 | 0.913 |
|  | Glucose | -0.024 | 0.551 | 0.872 |
|  | Triglycerides | 0.132 | 0.452 | 0.666 |
|  | Creatinine | -0.007 | 0.057 | 0.208 |
|  | Lipoprotein A | -0.023 | -0.263 | 0.648 |
|  | Apolipoprotein B | 0.038 | -0.388 | 0.632 |
|  | HDL cholesterol | 0.007 | -0.487 | 0.699 |
|  | Cholesterol | -0.093 | -0.606 | 0.912 |
| Alzheimer’s | Alzheimer’s disease | 10.719 | 0.793 | 0.245 |
|  | Other cerebrovascular | 0.508 | 0.151 | 0.154 |
|  | Apolipoprotein B | 0.017 | 0.125 | 0.060 |
|  | Cholesterol | 0.022 | 0.116 | 0.056 |
|  | Glucose | 0.029 | 0.092 | 0.053 |
|  | Type 2 diabetes mellitus | 0.699 | 0.075 | 0.064 |
|  | Triglycerides | 0.025 | 0.061 | 0.044 |
|  | Cerebral infarction | 0.244 | 0.058 | 0.150 |
|  | Acute ischemic heart disease | 0.684 | 0.015 | 0.159 |
|  | HbA1c | -0.017 | 0.007 | 0.043 |
|  | HDL cholesterol | 0.005 | -0.001 | 0.041 |
|  | Essential hypertension | -0.012 | -0.013 | 0.053 |
|  | Lipoprotein A | -0.001 | -0.022 | 0.142 |
|  | Creatinine | -0.040 | -0.062 | 0.044 |
|  | Obesity | -0.255 | -0.070 | 0.068 |
|  | Type 1 diabetes mellitus | -2.277 | -0.085 | 0.263 |
|  | Acute myocardial infarction | -0.485 | -0.100 | 0.068 |
|  | Ischemic heart disease | -0.173 | -0.103 | 0.063 |
|  | Other heart disease | -0.159 | -0.136 | 0.120 |
|  | Pulmonary heart diseases | -5.594 | -0.365 | 0.205 |
|  |  |  |  |  |
