# Supplementary material for: Optimized phenotype definitions boost GWAS power
Source: PLoS Comput Biol. 2026 Jul 1;22(7):e1014431. doi: 10.1371/journal.pcbi.1014431 (PMC13340767; doi:10.1371/journal.pcbi.1014431)
Supplement: Attachment — Submitted filename: Response to Reviewers_MaxGCP.docx [file pcbi.1014431.s007.docx]

Response to Reviewers

**Manuscript:** PCOMPBIOL-D-25-02439 **Title:** Optimized Phenotype Definitions Boost GWAS Power

Dear Editor and Reviewers,

We thank the editor and reviewers for their thoughtful evaluation of our manuscript. We have carefully considered each comment and provide detailed responses below. Reviewer comments are shown in **bold**, and our responses follow each point. We indicate where corresponding changes have been made in the revised manuscript.

Response to Editor / Journal Requirements

**1. CRediT author contributions for all co-authors.**

We have added complete CRediT author contributions for all authors in the revised submission form.

**2. Manuscript source file in .doc, .docx, .rtf, or .tex format.**

We now provide the manuscript as a LaTeX source file (.tex), uploaded under the item type ‘LaTeX Source File.’

**3. Author Summary (150–200 words) between Abstract and Introduction.**

We have added an Author Summary to the revised manuscript.

“Genome-wide association studies (GWAS) seek to identify genetic variants associated with disease risk. However, the phenotype definitions used in these studies—often derived from electronic health records—contain substantial environmental noise that reduces statistical power. We developed MaxGCP, a method that constructs optimized phenotype definitions by combining multiple observed phenotypes (e.g., diagnosis codes) into a single index that maximizes the shared genetic signal with a target disease. MaxGCP requires only summary-level genetic covariance estimates and a phenotypic covariance matrix, and it has an exact, closed-form solution that scales linearly with the number of input phenotypes. In simulations and real-data analyses of stroke and Alzheimer’s disease using the UK Biobank, MaxGCP consistently improved GWAS sensitivity compared to conventional single-code phenotype definitions, with the strongest gains observed when high-quality genetic covariance estimates were available. MaxGCP is freely available as a Python package and can be applied broadly to improve genetic discovery in observational data.”

**4. Main figures uploaded as separate .tif or .eps files.**

Done. All main figures have been converted and uploaded as separate files.

**5. Supplementary figures/tables removed from manuscript and uploaded as Supporting Information.**

Done. Supplementary materials are now uploaded separately with legends listed after the references in the main manuscript.

Response to Reviewer #1

We thank Reviewer #1 for an exceptionally detailed and constructive review. The depth of engagement with both the methodology and the evaluation framework has significantly improved the manuscript.

**Introduction**

**1. “Reference [3] is about warfarin dosing, which seems tangential. Consider a more direct citation.”**

We agree that Reference [3] (Sagreiya & Altman, 2010) is somewhat indirect, though it does demonstrate the general point that observational data quality affects study power. We have added a more directly relevant citation (Duan et al., AMIA 2017), which empirically demonstrates how measurement errors in EHR data reduce power in association studies. The original reference is retained as supporting evidence.

**2. “Overstated claims: ‘Unlike previous methods, MaxGCP is scalable, efficient, broadly-applicable, and does not require manual feature selection.’ MTAG is also scalable and broadly applicable.”**

We agree that this phrasing is imprecise. We have revised this sentence to more accurately describe the specific advantages of MaxGCP:

Abstract: “Unlike previous phenotype-combination methods, MaxGCP is phenotype-specific , has linear computational complexity in the number of features and does not require manual feature pre-selection.

**3. “The introduction should briefly mention what ‘coheritability’ means.”**

Good point. We have added a brief parenthetical definition upon first use:

“MaxGCP optimizes a phenotype definition to maximize its coheritability—the genetic covariance between two traits normalized by their phenotypic standard deviations—with the complex trait of interest.”

**4. “The transition from ‘previous methods require pre-selected features’ to ‘MaxGCP doesn’t’ is abrupt.”**

We have expanded this transition to explain the mechanism:

“Existing multi-trait methods such as MTAG are phenotype-specific but require pairwise LD score regressions among all features, resulting in quadratic computational complexity that limits scalability to large feature sets.”

“Because MaxGCP optimizes for genetic signal relevant to a specific target trait, irrelevant features receive near-zero weight automatically, eliminating the need for manual feature pre-selection.”

**Results – Section 2 (Overview)**

**“The mathematical setup could benefit from a conceptual explanation before the equations. Why does maximizing coheritability (rather than genetic correlation or heritability) make sense?”**

We have added the following conceptual motivation before the equations:

“We chose coheritability as the optimization objective because it directly reflects the quantity we seek to maximize in GWAS: the genetic signal relative to total phenotypic noise. Coheritability is proportional to genetic covariance and inversely proportional to phenotypic standard deviation, meaning that maximizing it simultaneously amplifies the shared genetic signal and suppresses environmental variance. By contrast, maximizing heritability alone would not ensure relevance to the target phenotype, and maximizing genetic correlation does not account for the overall noise level of the index.”

**Results – Section 2.1 (Simulation)**

**“Ground truth definition is circular—the genetic component GWAS defines perfect performance by construction.”**

We acknowledge this design choice and have clarified the rationale in the revised manuscript. The genetic component GWAS is used as a reference standard *precisely because* it isolates the true genetic signal from environmental noise. This is not circular in the traditional sense: we are not evaluating MaxGCP against itself, but rather against an oracle that defines the best possible outcome. The purpose is to measure how close each observable phenotype definition comes to this theoretical ideal, which is the standard approach in simulation studies where ground truth is known. We have added text noting that absolute power (number of true causal variants detected) is reported in the supplementary table (S1 Table) for completeness.

**“P-value threshold: Using p < 0.05 rather than genome-wide significance (5×10⁻⁸) makes the simulation less realistic.”**

We used p < 0.05 deliberately to ensure sufficient statistical events for a stable comparison of methods. At genome-wide significance (5×10⁻⁸), the simulation (10,000 individuals, 100,000 variants, 1% heritability) yields very few genome-wide significant hits for any method, making comparative evaluation unstable. We note that this threshold choice does not favor any particular method—it is applied uniformly across all comparisons. Moreover, as discussed in the manuscript, the majority of SNP heritability for complex traits is attributable to variants that do not reach genome-wide significance in current cohorts (Yang et al. 2010; Liu et al. 2019), so evaluation at lenient thresholds provides information about the sub-significant genetic architecture that drives polygenic risk. We have added clarifying language in Section 2.1 to make this rationale more explicit.

Additionally, in the real data analysis (Section 2.3, Figure 7), we evaluate performance across a range of p-value cutoffs from 0.05 to 5×10⁻⁸, and MaxGCP’s advantage is consistent throughout. This addresses the concern that our findings may not hold at stringent thresholds.

**“Table 1 vs Figure 2 redundancy: consider consolidating.”**

We have moved Table 1 to the supplement to reduce redundancy. Figure 2 provides the distributional information that the table summarizes, and we reference the supplementary table for readers who prefer exact values.

**“Missing: Effect size distributions of detected vs. missed variants.”**

This is an interesting question. In our simulation framework, all causal variants are assigned effect sizes drawn from the same distribution (as specified by PhenotypeSimulator), and MaxGCP operates on the phenotype definition rather than on individual variant associations. Therefore, MaxGCP does not selectively target larger or smaller effects—it uniformly amplifies the genetic signal component of the phenotype. We have added a sentence noting this in the revised text. An analysis stratifying detected variants by effect size would be informative but is beyond the scope of the current simulation design, where the number of causal variants per phenotype is relatively small.

Results – Section 2.2. (Real Data)

**“Low overall sensitivity (~12% at p<0.05) is concerning.”**

We agree this deserves prominent discussion and have expanded the relevant paragraph. The low absolute sensitivity reflects the fundamental challenge of replicating findings across different cohorts, genotyping platforms, and imputation pipelines—not a limitation specific to MaxGCP. Both the naive and MaxGCP phenotypes show low sensitivity because (1) the UK Biobank and reference GWAS (MEGASTROKE, IGAP) use different genotyping arrays and imputation strategies, leading to incomplete variant overlap; (2) the reference GWAS are far larger meta-analyses (MEGASTROKE: N=520k; IGAP: N=74k pooled across many cohorts) with correspondingly greater power; and (3) direct variant-level comparison is a conservative evaluation—colocalization-based approaches would likely show higher concordance. The key result is the *relative* improvement: MaxGCP nearly doubles sensitivity compared to naive definitions while maintaining comparable specificity, demonstrating that the method successfully amplifies genetic signal.

**“Specificity-sensitivity tradeoff: Is a 27% sensitivity gain worth a 1.7% specificity loss?”**

Whether this tradeoff is worthwhile depends on the research context. In genetic discovery settings—where the goal is to identify novel loci for downstream validation—sensitivity is typically prioritized over specificity, as false positives can be filtered through replication and fine-mapping. We have added a paragraph to the Discussion noting that MaxGCP is best suited for discovery-oriented analyses where identifying additional true signals outweighs the cost of modest increases in false positive rates. For confirmatory analyses where specificity is paramount, users may prefer conventional phenotype definitions or apply more stringent p-value cutoffs.

**“Validation against different populations: MEGASTROKE and IGAP include diverse ancestries, while MaxGCP was trained on White British UK Biobank participants.”**

We acknowledge this asymmetry. However, we note that this asymmetry works *against* MaxGCP’s apparent performance: if population stratification were inflating MaxGCP results, we would expect *higher* sensitivity and *lower* specificity than the true values. The fact that MaxGCP still outperforms naive definitions despite this ancestry mismatch suggests that the improvements are driven by genuine genetic signal rather than population stratification artifacts. We have added this reasoning to the Discussion and explicitly acknowledge the White British restriction as a limitation that should be addressed in future multi-ancestry analyses.

**“Feature selection: Why 50 features? Was this optimized or arbitrary?”**

The choice of 50 was pragmatic rather than optimized: we selected the most common ICD-10 codes to ensure reliable genetic covariance estimates. Genetic covariance estimation via LD score regression becomes unreliable for low-prevalence binary phenotypes (Bulik-Sullivan et al. 2015; Ge et al. 2017), and recent work on trait selection in multi-trait GWAS has shown that including genetically uninformative traits does not monotonically increase power (Julienne et al. 2024, *HGG Advances*). We have added these citations to the Methods (Section 4.4) to justify the threshold. We agree that a more comprehensive feature-selection analysis could be informative; however, our access period for UK Biobank data has ended, preventing us from conducting additional analyses at this stage.

MaxGCP is also inherently robust to the number of input features for two reasons. First, irrelevant features automatically receive near-zero weight because they have low genetic covariance with the target trait (as illustrated in Figure 6, where most features cluster near zero). Second, our quality control step—removing features with unreliable genetic covariance estimates—naturally limits the effective feature set. The MTAG comparison (Section 2.3) provides indirect evidence of this robustness: MaxGCP performed comparably with 5 features (the MTAG-compatible subset) and 50 features (the full set), suggesting that additional features neither help nor hurt substantially when genetic covariance estimates are reliable. We have added a paragraph in the Discussion noting this robustness and recommending that users include all features with reliable genetic covariance estimates rather than attempting to optimize the feature count.

**“Figure 4 interpretation: Panel B is more informative. Consider reordering.”**

We have reordered Figure 4 so that the fixed-threshold comparison (previously Panel B) is now presented first, as it provides the fairer comparison between methods.

Results – Section 2.3 (Comparison to MTAG)

**“Incomplete comparison: excludes Alzheimer’s and uses only 10 features.”**

We acknowledge these limitations, which stem from constraints of the MTAG software rather than our method. MTAG requires pairwise LD score regressions among all features (O(n²)), making it computationally infeasible for 50+ features, and it requires allele frequency information not available in the IGAP summary statistics. These are precisely the practical constraints that motivate MaxGCP’s design. We have clarified in the text that these limitations reflect MTAG’s requirements, not a deliberate choice to disadvantage MTAG, and we note that MaxGCP’s ability to scale to many features without these constraints is itself a key contribution.

**“Feature selection for MTAG: selecting by highest genetic correlation may disadvantage MTAG.”**

This is a fair point. We chose the top genetic correlation features because this is the most natural and principled selection criterion given the available information—features with high genetic correlation to the target are expected to be most informative for both methods. We are not aware of a recommended feature selection procedure specific to MTAG. We have added a note acknowledging that alternative feature selection strategies might yield different results for MTAG, though we note that this difficulty in feature selection is itself evidence of MTAG’s practical limitations compared to MaxGCP’s automatic approach.

**“Figure 7 complexity.”**

We have improved Figure 7 by adding panel labels, increasing font sizes, and adding a brief guide in the caption. We considered splitting the figure but believe that the side-by-side comparison across phenotypes is important for the reader to assess consistency.

Discussion

**1. “Overstated: ‘MaxGCP can nearly double sensitivity’ — both values are low.”**

We have revised this to: “In real data, MaxGCP approximately doubled GWAS sensitivity relative to naive phenotype definitions (e.g., from 6.5% to 12.0% for any stroke at p < 0.05). While both absolute values are low—reflecting the inherent difficulty of cross-cohort variant replication—the relative improvement demonstrates that MaxGCP successfully amplifies genetic signal.”

**2a. “When NOT to use MaxGCP.”**

We have added a paragraph to the Discussion:

“MaxGCP may not be appropriate in all settings. Specifically, we caution against using MaxGCP when (1) genetic covariance estimates are unreliable, such as when cohort sizes are small or when the feature phenotypes are rare, yielding noisy SumHer/LDSC estimates; (2) the target phenotype has low genetic correlation with available feature phenotypes, in which case MaxGCP has little information to exploit; or (3) the goal is confirmatory analysis where specificity is prioritized over sensitivity, as MaxGCP trades specificity for sensitivity gains.”

**2b. “Computational requirements: runtime and memory.”**

MaxGCP’s computational cost is dominated by the upstream estimation of genetic covariances (via SumHer/LDSC), not by the MaxGCP optimization itself. The optimization step—solving a linear system of dimension m (number of features)—completes in under one second for m = 50 on a standard laptop. The number of LD score regressions required scales as O(m) for MaxGCP versus O(m²) for MTAG. We have added a paragraph reporting these practical details. [See also response to Reviewer #2, Point 3.]

**2c. “Applicability beyond binary phenotypes.”**

MaxGCP is formulated for any phenotypes with estimable genetic and phenotypic covariances—it does not assume binary features or binary targets. The mathematical framework (Section 4) is written in terms of continuous covariance matrices and applies equally to continuous phenotypes (e.g., biomarker levels, quantitative traits). In the current study, we used binary ICD-10 code phenotypes because they are the most common phenotype representation in biobank EHR data. We have added a sentence noting this generality and identifying continuous trait applications as a natural extension.

**2d. “Multiple testing in PheWAS context.”**

MaxGCP produces a single optimized phenotype definition per target, so a MaxGCP-enhanced PheWAS would involve the same number of GWAS as a conventional PheWAS. Standard multiple testing corrections (Bonferroni, FDR) can be applied in the usual way. The optimization step is performed once per target phenotype and does not introduce additional hypothesis tests. We have added this clarification.

**3. “Indirect genetic effects / population stratification: what specific steps can users take?”**

We have expanded this paragraph with concrete recommendations:

“To mitigate such biases, we recommend several practical steps: (1) using LD score regression intercepts as a diagnostic, where large intercepts suggest confounding and the corresponding genetic covariance estimates should be down-weighted or excluded; (2) restricting to genetically homogeneous cohorts or including sufficient principal components as covariates in the upstream GWAS; (3) examining MaxGCP coefficients for biologically implausible features that may indicate confounding (e.g., socioeconomic proxies receiving large weights); and (4) comparing MaxGCP results across ancestry-stratified subgroups when possible.”

**4a. “Restriction to White British participants.”**

We have added this as an explicit limitation. The restriction was necessary to reduce population stratification in genetic covariance estimates from a single cohort. Extending MaxGCP to multi-ancestry cohorts is an important future direction that would require either ancestry-stratified analyses or cross-ancestry genetic covariance estimation methods.

**4b. “Reliance on ICD codes rather than richer phenotyping.”**

We used ICD codes because they are universally available in biobank-scale EHR data, making our results broadly reproducible. MaxGCP can accept any phenotype with estimable genetic covariance as a feature—lab values, medication records, NLP-derived phenotypes, or curated phenotype algorithms. We have noted this flexibility and identified richer feature sets as a direction for future improvement.

**4c. “No assessment of computational reproducibility (random seeds, etc.).”**

Our simulation used 100 replicate sets of 50 phenotypes each (5,000 total phenotypes), providing robust estimates of performance distributions. The simulation code and random seeds are available in the GitHub repository. For the real data analysis, no random seeds are involved—the method is deterministic given fixed inputs (genetic covariance estimates and phenotypic covariance matrix).

Methods

**Section 4.1: “Assumption of positive definiteness. What happens with collinear features? Regularization?”**

The phenotypic covariance matrix P is positive semi-definite by construction. In practice, P estimated from data is typically positive definite unless features are perfectly collinear (which would imply redundant phenotypes). In our analysis, the 50 ICD-10 code features are not perfectly collinear—while some codes are correlated (e.g., different stroke subtypes), none are exact linear combinations of others. If near-singularity were encountered, standard regularization approaches (e.g., ridge-type shrinkage: P + λI) could be applied. Our Python implementation uses numpy.linalg.solve, which will raise an error for singular matrices, alerting the user. We have added a note in the Methods discussing this assumption and the regularization fallback.

**Section 4.1: “Numerical stability for large P matrices.”**

For the feature counts used in this study (m ≤ 50), numerical stability is not a concern—the linear system is small. For much larger feature sets (m > 1000), we would recommend regularization or dimensionality reduction of the feature set. We note that the quality control step (removing features with noisy genetic covariance estimates) naturally limits m and reduces collinearity. We have added a brief note.

**Section 4.3: “Small simulation size.”**

The simulation was intentionally smaller than the UK Biobank to enable rapid iteration and comprehensive evaluation (100 replicate sets × 50 phenotypes = 5,000 phenotypes evaluated). Our goal was not to recapitulate the UK Biobank at full scale but to verify the correctness of the method, evaluate its behavior under controlled conditions, and establish performance bounds. The real data analysis on the full UK Biobank (N=342,350) serves as the large-scale validation. We have clarified this rationale in the text.

**Section 4.3: “Parameter choices not well-justified (1% heritability, 50% shared effects, 10% missingness).”**

These parameters were calibrated against real data. As described in Section 5.2 of the supplement, we estimated the heritability, genetic correlation, and phenotypic correlation of the 20 most common ICD-10 codes in the UK Biobank and set simulation parameters to produce distributions matching or more conservative than the real values (Figure S1). Specifically: median real heritability was 0.05 (we used 0.01 to be conservative); median real genetic correlation was 0.49 (we used 0.5); median real phenotypic correlation was 0.10 (we used 0.0 to be conservative). The 10% missingness rate reflects a realistic level of case under-ascertainment in EHR data. We have added a cross-reference to the supplement in Section 4.3 to make this justification more visible.

**Section 4.4: “Sample overlap between UK Biobank and MEGASTROKE/IGAP.”**

We have verified that the MEGASTROKE consortium explicitly excluded UK Biobank participants from their analysis, and the IGAP study predates UK Biobank data availability. We have added an explicit statement confirming no sample overlap.

**Section 4.4: “LD reference panel population mismatch.”**

The SumHer tagging files we used were computed from European-ancestry samples, consistent with our White British UK Biobank cohort. We used the pre-computed tagging files recommended by Speed and Balding (2019) for European-ancestry analyses. We have added this detail to the Methods.

Response to Reviewer #2

We thank Reviewer #2 for their constructive feedback and the recognition that the topic is timely and relevant.

**1. “More explicitly discuss risks of noisy/biased covariance estimation, particularly in smaller cohorts. Include experiments on additional phenotypes, or specify where MaxGCP is expected to be reliable.”**

We have substantially expanded the discussion of input quality dependence, including adding the “when NOT to use MaxGCP” guidance (see response to Reviewer #1, Discussion point 2a). Regarding additional phenotypes: our evaluation includes five distinct phenotypes (four stroke subtypes and Alzheimer’s disease) spanning different genetic architectures, prevalences, and external validation sources. We chose these phenotypes because high-quality, independent reference GWAS were available (MEGASTROKE and IGAP) that explicitly excluded UK Biobank participants, enabling rigorous validation. Extending to additional phenotypes would require identifying similarly high-quality, non-overlapping reference GWAS—a non-trivial requirement that limits the number of phenotypes suitable for this validation approach. We have added text noting that MaxGCP is expected to be most reliable for phenotypes that (1) have moderate-to-high genetic correlation with available feature phenotypes, (2) are evaluated in cohorts large enough for stable genetic covariance estimation (we recommend N > 50,000 based on our subsampling analysis), and (3) have features that pass standard quality control filters for genetic covariance estimates.

**2. “Justify how sensitivity/specificity thresholds relate to realistic GWAS scenarios.”**

We evaluate at multiple thresholds precisely because the “right” threshold depends on the application. The p < 0.05 threshold provides maximum statistical power for method comparison (more events = more stable estimates). The 90% specificity / 10% sensitivity thresholds (Figure 4B) represent a more realistic operating point: 90% specificity means roughly 10% of null variants are called significant, which is stringent enough to be informative while lenient enough to evaluate methods with limited sample sizes. In the MTAG comparison (Figure 7), we evaluate across the full range from p < 0.05 to p < 5×10⁻⁸, showing consistent MaxGCP advantages throughout. We have added a paragraph explaining this rationale and noting that in practice, users should apply standard genome-wide significance thresholds (5×10⁻⁸) and expect the relative advantages of MaxGCP to hold, as our multi-threshold analysis in Figure 7 demonstrates.

**3. “Include direct comparison of runtime or computational costs.”**

We have added a new paragraph in the Discussion comparing computational costs:

“In terms of computational requirements, MaxGCP’s optimization step is negligible: solving a linear system of dimension m (number of features) takes under one second for m = 50 on a standard laptop. The dominant cost is the upstream estimation of genetic covariances, which requires one LD score regression per feature for MaxGCP (O(m)) versus one per feature pair for MTAG (O(m²)). For m = 50 features, this translates to 50 regressions for MaxGCP versus 1,275 for MTAG. Each LD score regression takes approximately 5–10 minutes on a single CPU core, so the wall-clock time difference is substantial: ~4–8 hours for MaxGCP versus ~100–200 hours for MTAG (before parallelization). This O(m) vs O(m²) scaling is the primary reason we were unable to run MTAG with 50+ features in this study.”

The specific wall-clock times depend on hardware and parallelization, but the O(m) vs O(m²) scaling difference is the key point and holds regardless of implementation details.

**4. “Explain cross-phenotype performance variation.”**

We have added a paragraph:

“MaxGCP’s performance varied across phenotypes, with the strongest improvements observed for any stroke and ischemic stroke, and more modest gains for Alzheimer’s disease. Several factors likely contribute to this variation. First, the stroke phenotypes have higher genetic correlations with available feature phenotypes (e.g., cerebral infarction, ischemic heart disease, hypertension), providing MaxGCP with more informative signal to combine. Second, the genetic architecture of stroke subtypes involves substantial pleiotropy with cardiovascular risk factors that are well-represented among common ICD-10 codes, whereas Alzheimer’s disease has a more distinct genetic basis dominated by APOE locus, with fewer genetically correlated features in the available feature set. Third, stroke-related codes are more prevalent in the UK Biobank than Alzheimer’s-related codes, leading to more reliable genetic covariance estimates for stroke features.”

**5. “Conduct haplotype-level analyses or fine-mapping of newly identified peaks.”**

We appreciate this suggestion. However, the goal of the current study is to introduce and validate MaxGCP as a phenotyping method, not to perform genetic discovery for specific diseases. Fine-mapping and haplotype analysis of individual loci would constitute a separate follow-up study for each target phenotype. The Manhattan plot comparisons (Figures S2, S3) serve their intended purpose: demonstrating that MaxGCP peaks correspond to known target GWAS signals (MEGASTROKE, IGAP) rather than artifacts from dominant features (e.g., pulmonary heart disease or T2DM). A systematic fine-mapping analysis is an excellent direction for future work applying MaxGCP to specific diseases, and we have noted this in the revised Discussion.

**6. “Clarify whether the 10 features selected for the MTAG comparison are consistent with those receiving the highest weights in the unrestricted MaxGCP model.”**

This is a good question. The 10 features were selected based on absolute genetic correlation with the target (not MaxGCP coefficient magnitude), because this is a method-agnostic selection criterion that does not favor either MaxGCP or MTAG. In practice, these features substantially overlap with—but are not identical to—the features receiving highest MaxGCP weights in the unrestricted model. This is expected because MaxGCP coefficients are correlated with, but not identical to, genetic correlations (Figure 6; Pearson R = 0.54–0.94), since MaxGCP also accounts for phenotypic covariance structure. We have added a clarifying sentence and a supplementary comparison showing the overlap between the top-10 features by genetic correlation and by unrestricted MaxGCP coefficient magnitude.
