# Supplementary material for: Optimized phenotype definitions boost GWAS power
Source: PLoS Comput Biol. 2026 Jul 1;22(7):e1014431. doi: 10.1371/journal.pcbi.1014431 (PMC13340767; doi:10.1371/journal.pcbi.1014431)
Supplement: Peer review history

# Peer review: Decision Letter 0

**DOI:** 10.1371/journal.pcbi.1014431.r001

**Authors:** Lin Hou, Can Yang

3 Feb 2026

-->PCOMPBIOL-D-25-02439

OPTIMIZED PHENOTYPE DEFINITIONS BOOST GWAS POWER

PLOS Computational Biology

Dear Dr. Tatonetti,

Thank you for submitting your manuscript to PLOS Computational Biology. After careful consideration, we feel that it has merit but does not fully meet PLOS Computational Biology's publication criteria as it currently stands. Therefore, we invite you to submit a revised version of the manuscript that addresses the points raised during the review process.

Please submit your revised manuscript by Apr 05 2026 11:59PM. If you will need more time than this to complete your revisions, please reply to this message or contact the journal office at ploscompbiol@plos.org. When you're ready to submit your revision, log on to https://www.editorialmanager.com/pcompbiol/ and select the 'Submissions Needing Revision' folder to locate your manuscript file.

Please include the following items when submitting your revised manuscript:

* A letter that responds to each point raised by the editor and reviewer(s). You should upload this letter as a separate file labeled 'Response to Reviewers'. This file does not need to include responses to formatting updates and technical items listed in the 'Journal Requirements' section below.

* A marked-up copy of your manuscript that highlights changes made to the original version. You should upload this as a separate file labeled 'Revised Manuscript with Track Changes'.

* An unmarked version of your revised paper without tracked changes. You should upload this as a separate file labeled 'Manuscript'.

If you would like to make changes to your financial disclosure, competing interests statement, or data availability statement, please make these updates within the submission form at the time of resubmission. Guidelines for resubmitting your figure files are available below the reviewer comments at the end of this letter

We look forward to receiving your revised manuscript.

Kind regards,

Lin Hou

Academic Editor

PLOS Computational Biology

Can Yang

Section Editor

PLOS Computational Biology

**Journal Requirements:**

If the reviewer comments include a recommendation to cite specific previously published works, please review and evaluate these publications to determine whether they are relevant and should be cited. There is no requirement to cite these works unless the editor has indicated otherwise.

1) Please ensure that the CRediT author contributions listed for every co-author are completed accurately and in full.

At this stage, the following Authors/Authors require contributions: Kathleen LaRow Brown, Undina Gisladottir, Michael Zietz, and Nicholas P. Tatonetti. Please ensure that the full contributions of each author are acknowledged in the "Add/Edit/Remove Authors" section of our submission form.

The list of CRediT author contributions may be found here: https://journals.plos.org/ploscompbiol/s/authorship#loc-author-contributions

2) We ask that a manuscript source file is provided at Revision. Please upload your manuscript file as a .doc, .docx, .rtf or .tex. If you are providing a .tex file, please upload it under the item type u2018LaTeX Source Fileu2019 and leave your .pdf version as the item type u2018Manuscriptu2019.

3) Please provide an Author Summary. This should appear in your manuscript between the Abstract (if applicable) and the Introduction, and should be 150-200 words long. The aim should be to make your findings accessible to a wide audience that includes both scientists and non-scientists. Sample summaries can be found on our website under Submission Guidelines:

https://journals.plos.org/ploscompbiol/s/submission-guidelines#loc-parts-of-a-submission

4) Please upload all main figures as separate Figure files in .tif or .eps format. For more information about how to convert and format your figure files please see our guidelines:

https://journals.plos.org/ploscompbiol/s/figures

5) We notice that your supplementary Figures, and Tables are included in the manuscript file. Please remove them and upload them with the file type 'Supporting Information'. Please ensure that each Supporting Information file has a legend listed in the manuscript after the references list.

**Reviewers' comments:**

Reviewer's Responses to Questions

**Comments to the Authors:**

**Please note here if the review is uploaded as an attachment.**

Reviewer #1: This manuscript introduces MaxGCP, a statistical method designed to improve phenotype definitions for genome-wide association studies (GWAS) using observational data such as electronic health records. The core innovation is optimizing a linear combination of feature phenotypes to maximize "coheritability" with a target phenotype of interest, thereby amplifying genetic signal while reducing environmental noise.

Key methodological contributions:

• MaxGCP finds optimal coefficients (β) for combining multiple phenotypes by solving: β̂ = P⁻¹v / √(v⊺P⁻¹v), where P is the phenotypic covariance matrix and v is the vector of genetic covariances between features and target

• Unlike previous methods (e.g., MaxH, MTAG), MaxGCP is phenotype-specific, scalable (linear computational complexity in features), and does not require manual feature pre-selection

Evaluation approach:

1. Simulation study: 5,000 phenotypes simulated using real UK Biobank genotypes; compared MaxGCP to naive single-code phenotypes and theoretical bounds

2. Real data analysis: UK Biobank data (N=350k and N=15k) validated against external GWAS (MEGASTROKE for stroke; IGAP for Alzheimer's)

3. Comparison to MTAG: Head-to-head comparison showing similar or better performance with better computational efficiency

Key findings:

• In simulation, MaxGCP improved sensitivity (0.47 vs 0.28 for naive) with minimal specificity loss

• In real data (stroke), MaxGCP boosted sensitivity by >13% at equivalent specificity

• Performance depends critically on quality of genetic covariance estimates

• MaxGCP prioritizes biologically relevant features (e.g., heart disease for stroke, T2DM for Alzheimer's)

POINT-BY-POINT CRITIQUE AND COMMENTS

Introduction

Concerns:

1. Citation needed: "observational data bring additional challenges such as incompleteness, noise, and bias [2], which reduce study power [3]" — Reference [3] is about warfarin dosing, which seems tangential. Consider a more direct citation.

2. Overstated claims: "Unlike previous methods, MaxGCP is scalable, efficient, broadly-applicable, and does not require manual feature selection." MTAG is also scalable and broadly applicable; the key distinction is computational complexity (linear vs. quadratic) and phenotype-specificity. Rephrase to be more precise.

3. Missing context: The introduction should briefly mention what "coheritability" means, as it's a less common term than heritability or genetic correlation.

4. Gap in logic: The transition from "previous methods require pre-selected features" to "MaxGCP doesn't" is abrupt. Why can MaxGCP avoid this? (Answer: because it's phenotype-specific and uses QC filters instead of manual selection—but this isn't stated.)

Results Section

Section 2 (Overview):

• The mathematical setup is clear but could benefit from a conceptual explanation before the equations. Why does maximizing coheritability (rather than genetic correlation or heritability) make sense?

Section 2.1 (Simulation):

Concerns:

o Ground truth definition: "Positives were defined as variants simulated to be causal that were associated with nominal statistical significance (p < 0.05) in the genetic component GWAS." This is circular—the genetic component GWAS defines perfect performance by construction. While acknowledged, this limits interpretation. Consider also reporting absolute power (number of true causal variants detected) rather than only relative metrics.

o P-value threshold: Using p < 0.05 rather than genome-wide significance (5×10⁻⁸) makes the simulation less realistic for GWAS applications. The authors acknowledge this in the discussion but should address it more directly here.

o Table 1 vs Figure 2 redundancy: These present the same information; consider consolidating.

o Missing: Effect size distributions of detected vs. missed variants. Does MaxGCP preferentially detect larger effects, or does it uniformly improve detection?

Section 2.2 (Real Data):

Concerns:

o Low overall sensitivity: Even with MaxGCP, sensitivity is only ~12% at p<0.05 (Table 2). This means MaxGCP identifies only ~12% of variants from the reference GWAS. While the relative improvement over naive (~6.5%) is meaningful, the absolute performance is concerning. This limitation should be more prominently discussed.

o Specificity-sensitivity tradeoff: MaxGCP improves sensitivity but reduces specificity (Table 2). The authors claim "it increases sensitivity more than it reduces specificity," but the evidence is mixed. For example, in cardioembolic stroke (N=350k), sensitivity increases from 0.059 to 0.075 (+27%) while specificity decreases from 0.947 to 0.931 (-1.7%). Is a 27% sensitivity gain worth a 1.7% specificity loss? This depends on the application and should be discussed.

o Validation against different populations: MEGASTROKE and IGAP include diverse ancestries, while MaxGCP was trained on White British UK Biobank participants. Population stratification could affect the comparison.

o Feature selection: "We used the 50 most common ICD-10 codes" — Why 50? Was this optimized, or arbitrary? A sensitivity analysis varying the number of features would strengthen the paper.

o Figure 4 interpretation: Panel A shows raw sensitivity/specificity at p<0.05; Panel B shows sensitivity at 90% specificity and specificity at 10% sensitivity. The latter is more informative for fair comparison but is presented second. Consider reordering.

Section 2.3 (Comparison to MTAG):

Concerns:

o Incomplete comparison: MTAG comparison excludes Alzheimer's (due to missing allele frequencies in IGAP) and uses only 10 features (due to computational constraints). This significantly limits the comparison's generalizability.

o Feature selection for MTAG: "We selected the 10 features in decreasing order of their absolute genetic correlation to the target phenotype." This may disadvantage MTAG if its optimal features differ from those with highest genetic correlation. Did the authors consider alternative feature selection strategies?

o Figure 7 complexity: The figure is difficult to interpret with four methods across three phenotypes and two metrics. Consider simplifying or splitting.

Discussion

Concerns:

1. Overstated claims: "MaxGCP can nearly double sensitivity (Figure 4A)" — Figure 4A shows sensitivity of ~0.12 for MaxGCP vs ~0.06 for naive, which is technically "nearly double," but both values are low. The framing could be more measured.

2. Missing discussion points:

o When NOT to use MaxGCP: Under what conditions might naive phenotypes be preferable? (e.g., when genetic covariance estimates are unreliable, when phenotypes are not genetically correlated)

o Computational requirements: While MaxGCP is more efficient than MTAG, what are the actual runtime and memory requirements? This affects practical applicability.

o Applicability beyond binary phenotypes: Can MaxGCP handle continuous phenotypes? This is relevant for biomarkers and quantitative traits.

o Multiple testing: If MaxGCP is applied across many phenotypes in a PheWAS, how should multiple testing be handled?

3. Indirect genetic effects: The discussion of bias from population stratification (ZIP code example) is excellent but incomplete. What specific steps can users take to mitigate this? The suggestion of "penalizing genetic covariance estimates likely biased by population stratification" is too vague.

4. Missing limitations:

o Restriction to White British participants limits generalizability

o Reliance on ICD codes (rather than richer phenotyping) as features

o No assessment of computational reproducibility (random seeds, etc.)

Methods

Section 4.1 (Coheritability Optimization):

Concerns:

o Assumption of positive definiteness: The solution requires P to be invertible (positive definite). What happens when features are highly collinear? Is regularization needed?

o Numerical stability: For large P matrices, direct inversion may be numerically unstable. Do the authors use pseudoinverse or regularization in practice?

Section 4.3 (Simulation Analysis):

Concerns:

o Small simulation size: 10,000 individuals and 100,000 variants is much smaller than typical GWAS. While acknowledged, this limits interpretability.

o Parameter choices: Why 1% mean heritability, 50% shared genetic effects, 10% missingness? These seem reasonable but are not well-justified.

Section 4.4 (Real Data Analysis):

Concerns:

o Sample overlap: Were any UK Biobank participants in MEGASTROKE or IGAP? If so, this would inflate apparent performance. The authors should explicitly state that reference GWAS "do not include samples from the UK Biobank" (as mentioned in the text), but this is an assumption that should be verified.

o LD reference panel: SumHer uses pre-computed tagging files. Were these computed in the same population? Population mismatch could bias genetic covariance estimates.

Reviewer #2: This manuscript investigates an approach to boosting GWAS power by jointly modeling multiple phenotypes. The topic is timely and relevant, addressing an important challenge related to limited heritability and phenotypic noise in complex traits, and it fits well within the scope of the journal. While the study presents promising results and a theoretically elegant framework, several major issues need to be addressed before the manuscript can be considered for publication.

1. The performance of MaxGCP appears to be highly dependent on the quality of genetic covariance estimates. The authors are encouraged to more explicitly discuss the potential risks associated with noisy or biased covariance estimation, particularly in smaller cohorts. In addition, the current validation is somewhat limited. It would strengthen the manuscript to include experiments on additional complex phenotypes, or to clearly specify the types of phenotypes for which MaxGCP is expected to be reproducible and reliable.

2. The study evaluates performance using multiple sensitivity and specificity thresholds (e.g., 90% and 95% specificity). While this provides a comprehensive assessment, it would be helpful for the authors to justify how these thresholds relate to realistic GWAS application scenarios. In particular, clarification on how true positive and true negative trade-offs should be determined in practice would improve the interpretability of the results.

3. The manuscript reports that MaxGCP shows advantages over MTAG in terms of memory usage and performance under multiple evaluation criteria. To further substantiate the claimed computational advantages, it is recommended to include a direct comparison of runtime or computational costs. This would allow for a more comprehensive and intuitive demonstration of the benefits of MaxGCP in practical applications.

4. In the “real data” part of the result section, substantial differences were observed in the degree of improvement achieved by MaxGCP across the five phenotypes analyzed. An explicit discussion of the possible reasons for these differences—such as phenotypic heterogeneity, genetic architecture, or varying degrees of pleiotropy—would greatly enhance the biological and methodological interpretation of the results.

5. The authors state that “MaxGCP GWAS identified target peaks that are not present in the naive GWAS but are in the target GWAS” (Figures S2 and S3). To further validate these findings and to better characterize the genetic structure underlying these signals, it is recommended to conduct additional haplotype-level analyses or related fine-mapping investigations. These analyses would further strengthen the interpretation of the newly identified peaks.

6. In the statement “For this comparison, we used four different phenotype methods: the naive phenotype (a single ICD code), MTAG (with 10 features), MaxGCP (with 10 features), and MaxGCP (unrestricted features),” it would be helpful to clarify whether the selected 10 features are consistent with those receiving the highest weights in the unrestricted MaxGCP model.

**********

**Have the authors made all data and (if applicable) computational code underlying the findings in their manuscript fully available?**

The PLOS Data policy requires authors to make all data and code underlying the findings described in their manuscript fully available without restriction, with rare exception (please refer to the Data Availability Statement in the manuscript PDF file). The data and code should be provided as part of the manuscript or its supporting information, or deposited to a public repository. For example, in addition to summary statistics, the data points behind means, medians and variance measures should be available. If there are restrictions on publicly sharing data or code —e.g. participant privacy or use of data from a third party—those must be specified.

Reviewer #1: None

Reviewer #2: None

**********

PLOS authors have the option to publish the peer review history of their article (what does this mean?). If published, this will include your full peer review and any attached files.

If you choose “no”, your identity will remain anonymous but your review may still be made public.

**Do you want your identity to be public for this peer review?**  For information about this choice, including consent withdrawal, please see our Privacy Policy.

Reviewer #1: No

Reviewer #2: No

**Figure resubmission:**

While revising your submission, we strongly recommend that you use PLOS’s NAAS tool (https://ngplosjournals.pagemajik.ai/artanalysis) to test your figure files. NAAS can convert your figure files to the TIFF file type and meet basic requirements (such as print size, resolution), or provide you with a report on issues that do not meet our requirements and that NAAS cannot fix.-->

After uploading your figures to PLOS’s NAAS tool - https://ngplosjournals.pagemajik.ai/artanalysis, NAAS will process the files provided and display the results in the "Uploaded Files" section of the page as the processing is complete. If the uploaded figures meet our requirements (or NAAS is able to fix the files to meet our requirements), the figure will be marked as "fixed" above. If NAAS is unable to fix the files, a red "failed" label will appear above. When NAAS has confirmed that the figure files meet our requirements, please download the file via the download option, and include these NAAS processed figure files when submitting your revised manuscript.

**Reproducibility:**

To enhance the reproducibility of your results, we recommend that authors of applicable studies deposit laboratory protocols in protocols.io, where a protocol can be assigned its own identifier (DOI) such that it can be cited independently in the future. Additionally, PLOS ONE offers an option to publish peer-reviewed clinical study protocols. Read more information on sharing protocols at https://plos.org/protocols?utm_medium=editorial-email&utm_source=authorletters&utm_campaign=protocols

# Author comment: Author response to Decision Letter 1

**DOI:** 10.1371/journal.pcbi.1014431.r002

29 May 2026

Attachment Submitted filename: Response to Reviewers_MaxGCP.docx

# Peer review: Decision Letter 1

**DOI:** 10.1371/journal.pcbi.1014431.r003

**Authors:** Lin Hou, Can Yang

7 Jun 2026

Dear Dr Tatonetti,

We are pleased to inform you that your manuscript 'OPTIMIZED PHENOTYPE DEFINITIONS BOOST GWAS POWER' has been provisionally accepted for publication in PLOS Computational Biology.

Before your manuscript can be formally accepted you will need to complete some formatting changes, which you will receive in a follow up email. A member of our team will be in touch with a set of requests.

Please note that your manuscript will not be scheduled for publication until you have made the required changes, so a swift response is appreciated.

IMPORTANT: The editorial review process is now complete. PLOS will only permit corrections to spelling, formatting or significant scientific errors from this point onwards. Requests for major changes, or any which affect the scientific understanding of your work, will cause delays to the publication date of your manuscript.

Should you, your institution's press office or the journal office choose to press release your paper, you will automatically be opted out of early publication. We ask that you notify us now if you or your institution is planning to press release the article. All press must be co-ordinated with PLOS.

Thank you again for supporting Open Access publishing; we are looking forward to publishing your work in PLOS Computational Biology.

Best regards,

Lin Hou

Academic Editor

PLOS Computational Biology

Can Yang

Section Editor

PLOS Computational Biology

***********************************************************

Reviewer's Responses to Questions

**Comments to the Authors:**

**Please note here if the review is uploaded as an attachment.**

Reviewer #1: The authors have addressed my previous comments.

Reviewer #2: I have no comments. It can be accepted.

**********

**Have the authors made all data and (if applicable) computational code underlying the findings in their manuscript fully available?**

The PLOS Data policy requires authors to make all data and code underlying the findings described in their manuscript fully available without restriction, with rare exception (please refer to the Data Availability Statement in the manuscript PDF file). The data and code should be provided as part of the manuscript or its supporting information, or deposited to a public repository. For example, in addition to summary statistics, the data points behind means, medians and variance measures should be available. If there are restrictions on publicly sharing data or code —e.g. participant privacy or use of data from a third party—those must be specified.

Reviewer #1: Yes

Reviewer #2: None

**********

PLOS authors have the option to publish the peer review history of their article (what does this mean?). If published, this will include your full peer review and any attached files.

If you choose “no”, your identity will remain anonymous but your review may still be made public.

**Do you want your identity to be public for this peer review?**  For information about this choice, including consent withdrawal, please see our Privacy Policy.

Reviewer #1: No

Reviewer #2: No

# Editor report: Acceptance letter

**DOI:** 10.1371/journal.pcbi.1014431.r004

**Authors:** Lin Hou, Can Yang

PCOMPBIOL-D-25-02439R1

OPTIMIZED PHENOTYPE DEFINITIONS BOOST GWAS POWER

Dear Dr Tatonetti,

I am pleased to inform you that your manuscript has been formally accepted for publication in PLOS Computational Biology. Your manuscript is now with our production department and you will be notified of the publication date in due course.

The corresponding author will soon be receiving a typeset proof for review, to ensure errors have not been introduced during production. Please review the PDF proof of your manuscript carefully, as this is the last chance to correct any errors. Please note that major changes, or those which affect the scientific understanding of the work, will likely cause delays to the publication date of your manuscript.

Soon after your final files are uploaded, unless you have opted out, the early version of your manuscript will be published online. The date of the early version will be your article's publication date. The final article will be published to the same URL, and all versions of the paper will be accessible to readers.

For Research, Software, and Methods articles, you will receive an invoice from PLOS for your publication fee after your manuscript has reached the completed accept phase. If you receive an email requesting payment before acceptance or for any other service, this may be a phishing scheme. Learn how to identify phishing emails and protect your accounts at https://explore.plos.org/phishing.

Thank you again for supporting PLOS Computational Biology and open-access publishing. We are looking forward to publishing your work!

With kind regards,

Zsofia Freund

PLOS Computational Biology | Carlyle House, Carlyle Road, Cambridge CB4 3DN | United Kingdom ploscompbiol@plos.org | Phone +44 (0) 1223-442824 | ploscompbiol.org | @PLOSCompBiol
